# Support for e-cigarette policies: a survey of smokers and ex-smokers in Great Britain

**DOI:** 10.1136/tobaccocontrol-2016-052987

**Published:** 2016-06-16

**Authors:** Leonie S Brose, Timea R Partos, Sara C Hitchman, Ann McNeill

**Affiliations:** Department of Addictions, Institute of Psychiatry, Psychology and Neuroscience, King's College London, UK and UK Centre for Tobacco and Alcohol Studies (UKCTAS), London, UK

**Keywords:** Electronic nicotine delivery devices, Public opinion, Public policy, Advertising and Promotion

## Abstract

**Introduction:**

E-cigarette regulations are the topic of extensive debate. Approaches vary worldwide, and limited evidence is available on public support for specific policies or what influences support. The present study aimed to assess smokers' and ex-smokers' support for 3 e-cigarette policies: (1) equal or higher availability relative to cigarettes, (2) advertising, (3) use in smoke-free places, and to assess changes in support over time and associations with respondent characteristics.

**Methods:**

Smokers and ex-smokers (n=1848) provided 3279 observations over 2 waves (2013 and 2014) of a longitudinal web-based survey in Great Britain. Multivariable logistic regressions fitted using generalised estimating equations assessed change in policy support over time, and associations between support and demographics (age, gender and income), smoking and e-cigarette use status, nicotine knowledge and perceived relative harm.

**Results:**

Equal or higher relative availability was supported by 79% in 2013 and 76% in 2014; advertising by 66% and 56%, respectively; neither change was significant in adjusted analyses. Support for use in smoke-free places decreased significantly from 55% to 45%. Compared with ex-smokers, smokers were more likely to support advertising and use in smoke-free places. Respondents using e-cigarettes, those who perceived e-cigarettes as less harmful than cigarettes, and those with more accurate knowledge about nicotine were more likely to support all 3 policies.

**Conclusions:**

Less restrictive e-cigarette policies were more likely to be supported by e-cigarette users, and respondents who perceived e-cigarettes to be less harmful than cigarettes, or knew that nicotine was not a main cause of harm to health.

## Introduction

E-cigarettes use battery power to heat a solution of propylene glycol or glycerine, water, flavouring and often nicotine, resulting in an aerosol that can be inhaled by the user (commonly termed vapour). In contrast with traditional cigarettes, e-cigarettes do not contain tobacco, do not create smoke and do not rely on combustion.^1^ Although the main reason for addiction to smoking is nicotine, the health harms of smoking are caused by other constituents of cigarette smoke.^2^ ^3^

Appropriate regulation of e-cigarettes is the topic of extensive debate, and approaches vary across countries.^4^ To date, it is not yet clear as to which e-cigarette policies will lead to desirable public health outcomes such as decreased harm from smoking. In traditional tobacco control, public support has been an important component in getting policies adopted in law.^5^ As there is much debate around the evidence base regarding e-cigarettes, it is important to understand people's views and the correlates of policy support. Three key domains of e-cigarette regulations are now discussed.

### Availability

A recent review of global approaches to regulation found that 21 countries restricted the sale of e-cigarettes with nicotine and 26 countries banned sale of all types of e-cigarettes.^4^ E-cigarettes are widely available in the UK; manufacturers may apply for medicinal licensing,^6^ or sell their e-cigarettes as unlicensed products under general product safety regulations, and from May 2016 under the revised European Union Tobacco Products Directive (TPD).^7^ In the UK, the Royal Society for Public Health has called for making e-cigarettes and other nicotine-containing products more widely available than combustible tobacco products.^8^ Their rationale was that making less harmful nicotine sources more easily available than cigarettes may encourage smokers to quit smoking and help those trying to quit smoking to overcome cravings.^8^ To date, there is no published evidence from the UK or elsewhere, on public preferences for availability of e-cigarettes.

### Advertising

The majority of countries that ban or restrict sale of e-cigarettes also prohibit or restrict advertising, promotion or sponsorship.^4^ In the European Union, from 2016, the TPD will lead to prohibition of advertising on broadcast and on demand TV, radio, print magazines and newspapers, internet, email, text message, sponsorship of activity or individuals with cross-border effects.^7^ ^9^ In the UK, current rules stipulate that e-cigarette advertising must not cross-promote tobacco brands, promote tobacco products and must make clear that the product is not a tobacco product.^10^ Advertising also must not be likely to appeal particularly to people under 18 years, nor show people who are or look under 25 years using e-cigarettes or playing a significant role.^10^ ^11^ Two surveys, both in the USA, have assessed aspects of support for advertising regulations.^12^ ^13^ One found lower support for advertising restrictions among smokers who had tried or used e-cigarettes, and smokers who believed e-cigarettes to be less harmful than cigarettes in unadjusted analyses;^12^ the other found universally high support for banning advertising e-cigarettes to youth under 18 years.^13^

### Use in smoke-free places

Policies banning tobacco smoking, for example, in public places or workplaces are based on the evidence that secondhand smoke exposure in enclosed public spaces is harmful to health.^14^ ^15^ There is little evidence that e-cigarette vapour/aerosol causes harm to the health of bystanders.^1^ Evidence does suggest that e-cigarette use increases the concentration of fine particulate matter suspended in air.^16^ ^17^ However, the composition of the particulate matter is different from cigarette smoke,^18^ and concentrations are far lower than those caused by cigarette smoke,^19^ and not always distinguishable from those in non-smoking and non-vaping environments.^20^ A small number of countries ban all use of e-cigarettes, some ban use in certain or all enclosed public spaces, others specifically prohibit use on public transportation.^4^ However, policies can also vary within countries. For example, in the UK, one of the four member countries has consulted on banning e-cigarette use in public places,^21^ while the other three UK countries do not have any plans to do so. Similarly, regulations differ between individual states within other countries, for example, in the USA or Australia. Often, policies are determined at a local level by local governments, companies or institutions, resulting in varying approaches.^22–24^ Previous surveys, one from Spain, the others from the USA, have assessed support for restrictions on use of e-cigarettes in enclosed public places or areas where smoking is not allowed.^12^ ^13^ ^25–29^ They generally found that those who had used or tried e-cigarettes, as well as smokers, were less supportive of restrictions. Perceiving e-cigarettes as less harmful than smoking was also associated with being less supportive of restrictions,^12^ while perceived harmfulness of breathing secondhand vapour^26^ ^27^ and perceived addictiveness of e-cigarettes^26^ were associated with higher support for restrictions.

### Aims

The present study aimed to answer the following research question: in a cohort of smokers and ex-smokers, how did support for different e-cigarette policies change over time, and how did it vary with sociodemographic characteristics, e-cigarette use, smoking status, perceived relative harm of e-cigarettes, and knowledge about what portion of health harms of smoking comes from nicotine? Support was assessed for policies on e-cigarette availability, advertising and use in smoke-free places in 2013 and 2014.

## Methods

### Design and sample

We used data from a longitudinal web-based survey of a general population sample of smokers and ex-smokers (ex-smokers had quit smoking during the year before the baseline wave) in Great Britain. Members of an online panel managed by Ipsos MORI were invited to participate in a survey about smoking. Members of the panel consent to complete surveys, and for each completed survey earn points which can be redeemed against shopping vouchers or used to enter a prize draw. Those who accepted the invitation (n=23 785) were screened, and past-year smokers (n=6165) were eligible for the survey. Quotas were imposed to ensure broad representativeness of the British population by gender, age and region at recruitment. Baseline/wave 1 (November/December 2012) was completed by 5000 respondents. The cohort data have previously been used to show that changes in smoking behaviour vary with the frequency of e-cigarette use^30^ and the type of device used,^31^ and to demonstrate an increase in the perceived harm of e-cigarettes relative to cigarettes over time.^32^ The present analyses include data from wave 2 in 2013 and wave 3 in 2014, when policy questions were added to the survey. In 2013, 2182 respondents completed the survey (43.6% of wave 1); n=93 not aware of e-cigarettes were not asked about e-cigarette policies, and n=198 ex-smokers who had quit smoking more than 1 year ago were erroneously not asked about e-cigarette policies. Wave 3 was completed by 1519 respondents (69.6% of wave 2), of whom n=42 not aware of e-cigarettes were not asked about e-cigarette policies. Respondents who did not know or disclose key information (other than income) were excluded (n=43), leaving n=1848 respondents who provided 3279 observations.

### Measures

#### Demographics

Demographics at wave 2 (2013) were used for analyses. They included age (for analysis, grouped as 18–24 years; 25–39 years; 40–54 years; 55 years and over) and gender (male and female). The analyses included annual non-equivalised household income (Under £6500; £6500–15 000; £15 001–30 000; £30 001–40 000; £40 001–50 000; £50 001–65 000; £65 001–95 000; £95 001 and over; ‘Don’t know’; ‘Prefer not to say’). The UK government defines ‘low income’ to be 40% below the national median income. In 2013/2014, this threshold was just over £14 000,^33^ which is why responses were collapsed into ‘up to £15000’ (low income), ‘£15001 to £30000’ (middle income) and ‘over £30000’ (higher income); owing to a considerable proportion of respondents selecting ‘don't know’ or ‘prefer not to say’, these responses were kept as an additional category. Since income could not be equivalised by household size, sensitivity analyses were also run using highest level of education (dichotomised into ‘no higher education’ and ‘higher education’) in its place.

#### E-cigarette use and smoking status

E-cigarette use status at each wave was derived from two questions: (1) ‘Have you ever tried an electronic cigarette? (a) Yes; (b) No; (c) Don't know’ and (2) ‘How often, if at all, do you currently use an electronic cigarette? (a) Daily; (b) Less than daily, but at least once a week; (c) Less than weekly, but at least once a month; (d) Less than monthly; (e) Not at all; (f) Don’t know’. Responses were combined to derive the following categories: Never tried (1b); tried, but not currently using (1a in combination with 2e); current non-daily use (1a in combination with 2b, 2c or 2d); current daily use (1a in combination with 2a). A small number of don't know responses were excluded (table 1). Smoking status was determined using the question: ‘Which of the following best applies to you? (a) I smoke cigarettes (including hand rolled) everyday; (b) I smoke cigarettes (including hand rolled) but not every day; (c) I do not smoke cigarettes at all but I do smoke tobacco of some kind (eg, pipe or cigar); (d) I have stopped smoking completely in the last year; (e) I stopped smoking more than a year ago; (f) Don't know/couldn't say’.^34^ For analysis, responses were collapsed into current smoker (a–c) or ex-smoker (d, e); (f) was excluded.

**Table 1:** Sample, longitudinal survey

	2013 n=1848		2014 n=1431		
	Frequency	Per cent	Frequency	Per cent	Comparison
Gender					χ^2^=0.69, p=0.41
Male	1084	58.7	860	60.1	
Female	764	41.3	571	39.9	
Age (years)					χ^2^=14.19, p=0.003
18–24	139	7.5	86	6.0	
25–39	471	25.5	314	21.9	
40–54	594	32.1	450	31.4	
55 and over	644	34.8	581	40.6	
Annual income					χ^2^=0.07, p>0.99
Up to £15 000 (low)	441	23.9	339	23.7	
£15 001–£30 000 (middle)	572	31.0	439	30.7	
Over £30 000 (high)	649	35.1	508	35.5	
Don't know/prefer not to say	186	10.1	145	10.1	
Smoking status					χ^2^=80.37, p<0.001
Ex-smoker	247	13.4	368	25.7	
Current smoker	1601	86.6	1063	74.3	
E-cigarette use status					χ^2^=7.29, p=0.06
Never tried	781	42.3	593	41.4	
Tried, not using	385	20.8	342	23.9	
Less than daily use	448	24.2	303	21.2	
Daily use	234	12.7	193	13.5	
Health risks of smoking from nicotine					χ^2^=3.65, p=0.60
None or very small	212	11.5	156	10.9	
Some but well under half the risk	452	24.5	378	26.4	
Around half the risk	393	21.3	314	21.9	
Much more than half the risk	359	19.4	280	19.6	
Nearly all the risk	231	12.5	168	11.7	
Don't know	201	10.9	135	9.4	
Relative harm of e-cigarettes					χ^2^=39.13, p<0.001
More harmful than cigarettes	38	2.1	32	2.2	
Equally harmful	199	10.8	263	18.4	
Less harmful than cigarettes	1199	64.9	843	58.9	
Don't know	412	22.3	293	20.5	

#### Nicotine knowledge and relative harm

Nicotine knowledge was measured at waves 2 and 3 by asking: ‘According to what you know or believe, what portion of the health risks of smoking comes from nicotine in cigarettes? (a) None or very small; (b) Some but well under half the risk; (c) Around half the risk; (d) Much more than half the risk; (e) Nearly all the risk; (f) Don't know’. Perceived relative harm was measured at waves 2 and 3 asking: ‘Do you think electronic cigarettes are more harmful than regular cigarettes, less harmful, or are they equally harmful to health? (a) More harmful than regular cigarettes; (b) Equally harmful; (c) Less harmful than regular cigarettes; (d) Don't know’. For analysis, the response options were dichotomised into less harmful (c) and all other, inaccurate, responses (a, b, d).

#### Policy support

Availability: ‘Do you think that electronic cigarettes should be: (a) As freely available and accessible as traditional cigarettes; (b) More available than traditional cigarettes; (c) Less available than traditional cigarettes; (d) Don't know’. For logistic regressions, these were collapsed into equal or higher availability relative to cigarettes (a, b) and all other responses (c, d).

Advertising: ‘Do you think that electronic cigarettes companies should be allowed to advertise e-cigarettes? (a) No, they should not be allowed to advertise electronic cigarettes (similar to traditional cigarettes); (b) Yes, they should be allowed to advertise electronic cigarettes, but not in a way that could attract children; (c) Yes, they should be allowed to advertise electronic cigarettes with no restrictions; (d) Don't know’. For logistic regressions, these were collapsed into ‘yes, should be allowed’ (b, c) vs should not be allowed/don't know (a, d).

Use in smoke-free places: ‘Do you think that people should be allowed to use electronic cigarettes in places where smoking is not allowed? (a) Yes; (b) No; (c) Don't know’; for logistic regressions, (b) and (c) were combined.

### Analysis

Changes in the composition of the sample from 2013 to 2014 were assessed using χ^2^ statistics. Taking into consideration the correlated nature of the data within respondents across survey waves, we used logistic regressions fitted using the generalised estimating equations approach to compute parameter estimates using an unstructured within-subject correlation structure which makes no assumption about the magnitude of the correlation between pairs of observations. We assessed the association between e-cigarette use status, smoking status, nicotine knowledge, relative harm, age, gender and annual income, and support for each of the three policies. In a first step, bivariate associations between each variable and policy were estimated; a second step estimated associations while adjusting for all other variables and included interactions between time-invariant demographics and wave. Sensitivity analyses replaced income with education. Education did not have a significant association with any of the outcomes, and did not substantively alter the associations between the outcomes and other predictors, so only the results using income will be presented here. All analyses were conducted using SPSS V.22.0.

## Results

### Sample and attrition

The sample included at each wave is described in table 1. The sample contained more men than women, a small proportion of young respondents, and almost a quarter on a low income; 63% had no higher education. The majority were smokers, and over a third of the sample was using e-cigarettes less than daily or daily. Only around 1 in 10 respondents knew that a very small portion of the health risks of smoking come from nicotine in smoke; a similar proportion thought that nearly all the health risks were caused by nicotine. A small majority perceived e-cigarettes to be less harmful than cigarettes. The sample in 2014 contained a higher proportion of ex-smokers than the 2013 sample, and had lost a higher proportion of younger respondents (mean age increased by about 2 years, so the change in the proportion in different age brackets is not fully explained by ageing).

### Policy support

#### Availability

**Figure 1:**
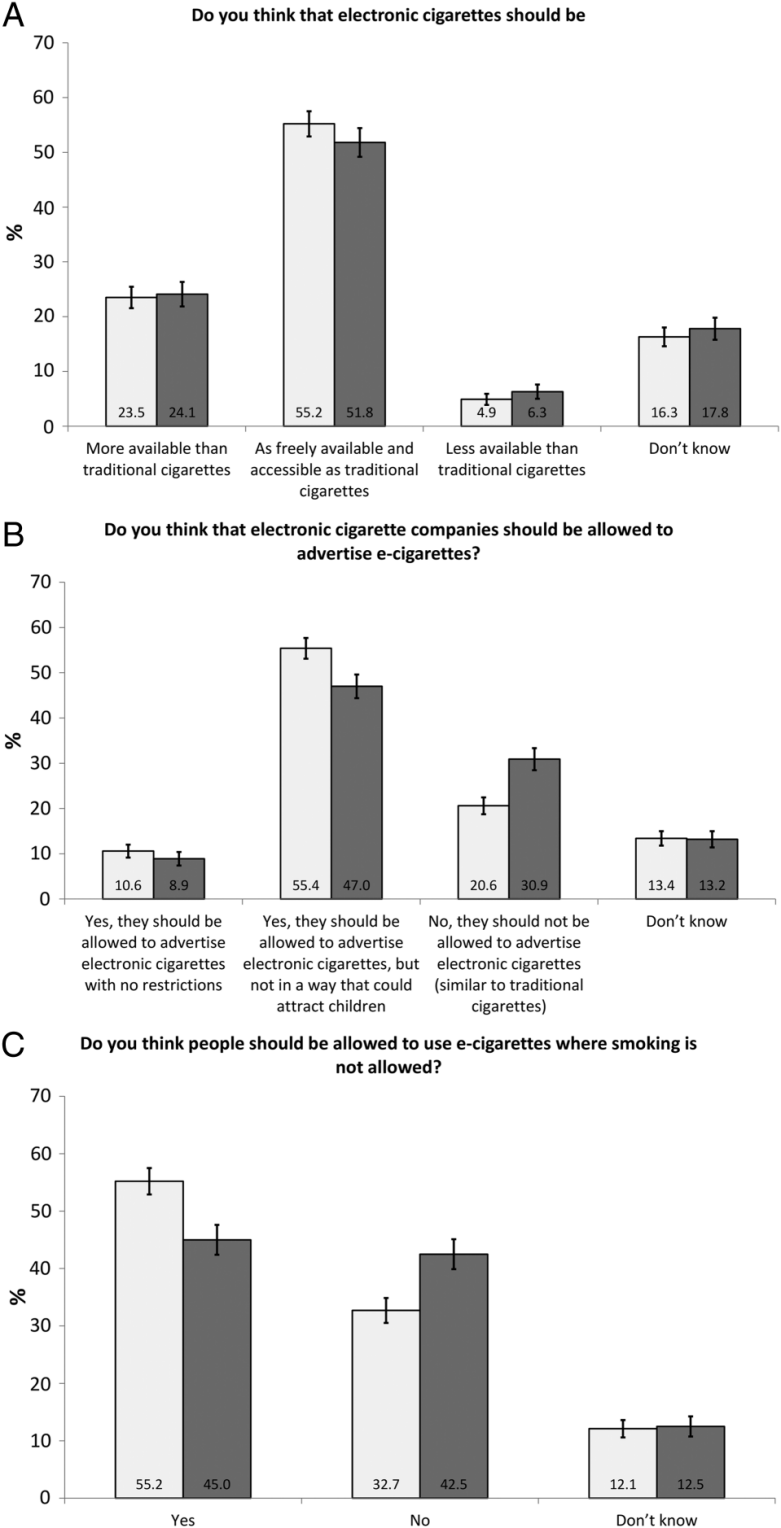
Support for regulations on (A) e-cigarette availability, (B) advertising and (C) use in smoke-free places. (2013: n=1848; 2014: n=1431).

Support for e-cigarettes being equally or more available than cigarettes decreased slightly over time from 78.8% in 2013 to 75.9% in 2014 (figure 1 and table 2), but this change was not significant in adjusted analysis. Only around 5% of respondents thought e-cigarettes should be less available than traditional cigarettes, with the remainder (16% and 18%, respectively) being undecided. Very similar proportions of ex-smokers and smokers thought that e-cigarettes should be equally or more available than cigarettes. Non-daily and daily e-cigarette users were more likely to support equal or higher availability than those who had never tried e-cigarettes in adjusted analysis. Compared with those who thought that nearly all the health risks of smoking came from nicotine, all other respondents were more likely to support equal or higher availability except for those who did not know what portion of risk was due to nicotine—this group was less likely to support equal or higher availability. Those who perceived e-cigarettes to be less harmful than cigarettes were far more likely to support equal or higher availability. Only in unadjusted analysis were those with a high income more likely, and those who did not disclose their income less likely to support equal or higher availability than those with a low income; age and gender were not associated with support (table 2). There was a significant interaction between gender and survey wave, suggesting that women were more likely to change from supporting to not supporting availability between waves than men (see online supplementary table S1).

**Table 2:** Association between respondent characteristics and support for availability of e-cigarettes relative to regular cigarettes

		Bivariate				Adjusted/Multivariable			
	Should be equally or more available (%)	OR	95% LCI	95% UCI	p Value	OR	95% LCI	95% UCI	p Value
Wave									
2013 (referent)	78.8	1.00	ref	ref	ref	1.00	ref	ref	ref
2014	75.9	0.85	0.74	0.98	**0.024**	1.07	0.50	2.27	0.86
Smoking status									
Ex-smoker (referent)	76.9	1.00	ref	ref	ref	1.00	ref	ref	ref
Current smoker	77.7	1.02	0.82	1.27	0.85	1.10	0.87	1.41	0.43
E-cigarette					**<0.001**				**<0.001**
Never tried (referent)	70.5	1.00	ref	ref	ref	1.00	ref	ref	ref
Tried, not using	75.9	**1.28**	**1.04**	**1.58**	**0.021**	1.08	0.86	1.35	0.52
Non-daily use	83.9	**2.08**	**1.65**	**2.61**	**<0.001**	**1.41**	**1.08**	**1.84**	**0.011**
Daily use	91.8	**4.61**	**3.22**	**6.58**	**<0.001**	**2.56**	**1.78**	**3.96**	**<0.001**
Risks of smoking from nicotine					**<0.001**				**<0.001**
Nearly all (referent)	72.9	1.00	ref	ref	ref	1.00	ref	ref	ref
Much more than half	78.6	1.27	0.95	1.70	0.12	**1.49**	**1.08**	**2.07**	**0.016**
Around half	78.8	**1.37**	**1.02**	**1.84**	**0.034**	**1.48**	**1.07**	**2.04**	**0.017**
Some but well under half	82.2	**1.67**	**1.25**	**2.24**	**0.001**	**1.47**	**1.06**	**2.02**	**0.019**
None/very small	85.9	**2.10**	**1.46**	**3.03**	**<0.001**	**1.50**	**1.01**	**2.24**	**0.046**
Don’t know	57.7	**0.52**	**0.38**	**0.71**	**<0.001**	**0.63**	**0.45**	**0.90**	**0.010**
Relative harm of e-cigarettes									
Not less harmful (referent)	56.4	1.00	ref	ref	ref	1.00	ref	ref	ref
Less harmful	90.3	**6.67**	**5.51**	**8.06**	**<0.001**	**5.66**	**4.66**	**6.88**	**<0.001**
Gender									
Male (referent)	78.8	1.00	ref	ref	ref	1.00	ref	ref	ref
Female	76.3	0.89	0.74	1.07	0.20	1.13	0.88	1.46	0.35
Age (years)					0.092				0.122
18–24 (referent)	79.9	1.00	ref	ref	ref	1.00	ref	ref	ref
25–39	74.3	0.69	0.47	1.03	0.066	0.68	0.40	1.17	0.16
40–54	78.1	0.87	0.59	1.28	0.47	1.01	0.60	1.72	0.96
55 and over	78.6	0.89	0.61	1.31	0.55	1.13	0.67	1.92	0.65
Annual income					**<0.001**				**0.25**
Up to £15 000 (referent)	76.0	1.00	ref	ref	ref	1.00	ref	ref	ref
£15 001–£30 000	78.3	1.13	0.88	1.43	0.34	0.98	0.70	1.37	0.91
Over £30 000	80.4	**1.30**	**1.02**	**1.66**	**0.035**	1.15	0.82	1.61	0.43
Don’t know/prefer not to say	68.6	**0.67**	**0.49**	**0.92**	**0.012**	0.82	0.54	1.23	0.33

Models include 3279 observations from 1848 individuals. Bold font indicates significant associations (p<0.05).

LCI, Lower CI; UCI, Upper CI.

10.1136/tobaccocontrol-2016-052987.supp1 Supplementary table S1 Interactions

#### Advertising

Support for allowing advertising was significantly lower in 2014 (55.9%) than in 2013 (66.0%) in the unadjusted analysis (figure 1 and table 3), but this change was no longer significant in the adjusted analysis (table 3). Most of those supporting advertising thought that advertising should be allowed, but in a way that would not attract children. In 2013, 20.6% and in 2014, 30.9% supported no restrictions on e-cigarette advertising, and at both waves, about 13% responded ‘don't know’ (figure 1). Compared with ex-smokers, smokers were more likely to respond that advertising should be allowed. The more experience respondents had with e-cigarettes, the higher the odds that they responded that advertising should be allowed. Compared with those who thought that nearly all the health risks of smoking came from nicotine, those who responded that it was none or a very small part, or well under half the risk (in adjusted analysis also those who responded ‘much more than half the risk’), were more likely to think that advertising should be allowed. Respondents perceiving e-cigarettes as less harmful than cigarettes were also more likely to support advertising. Age, gender and income were not associated with support (table 3). There were no significant interactions between demographics and survey wave suggesting that these associations did not change over time (see online supplementary table S1).

**Table 3:** Association between respondent characteristics and support for advertising of e-cigarettes

		Bivariate				Adjusted/multivariable			
	Advertising should be allowed (%)	OR	95% LCI	95% UCI	p Value	OR	95% LCI	95% UCI	p Value
Wave									
2013 (referent)	66.0	1.00	ref	ref	ref	1.00	ref	ref	ref
2014	55.9	**0.64**	**0.57**	**0.73**	**<0.001**	0.80	0.44	1.46	0.47
Smoking status									
Ex-smoker (referent)	56.1	1.00	ref	ref	ref	1.00	ref	ref	ref
Current smoker	62.9	**1.31**	**1.09**	**1.58**	**0.004**	**1.32**	**1.08**	**1.62**	**0.008**
E-cigarette					**<0.001**				**<0.001**
Never tried (referent)	52.7	1.00	ref	ref	ref	1.00	ref	ref	ref
Tried, not using	60.0	**1.35**	**1.12**	**1.62**	**0.002**	**1.23**	**1.01**	**1.50**	**0.043**
Non-daily use	69.9	**1.90**	**1.57**	**2.31**	**<0.001**	**1.44**	**1.17**	**1.79**	**0.001**
Daily use	78.5	**3.14**	**2.42**	**4.07**	**<0.001**	**2.20**	**1.65**	**2.93**	**<0.001**
Risks of smoking from nicotine					**<0.001**				**<0.001**
Nearly all (referent)	57.1	1.00	ref	ref	ref	1.00	ref	ref	ref
Much more than half	62.6	1.22	0.95	1.57	0.12	**1.35**	**1.03**	**1.77**	**0.031**
Around half	60.4	1.11	0.86	1.43	0.41	1.18	0.90	1.54	0.24
Some but well under half	66.4	**1.43**	**1.12**	**1.83**	**0.005**	**1.32**	**1.01**	**1.72**	**0.042**
None/very small	73.6	**1.88**	**1.39**	**2.56**	**<0.001**	**1.49**	**1.07**	**2.06**	**0.017**
Don’t know	42.6	**0.59**	**0.44**	**0.80**	**0.001**	**0.70**	**0.51**	**0.96**	**0.027**
Relative harm of e-cigarettes									
Not less harmful (referent)	39.6	1.00	ref	ref	ref	1.00	ref	ref	ref
Less harmful	74.9	**4.14**	**3.53**	**4.86**	**<0.001**	**3.55**	**3.00**	**4.20**	**<0.001**
Gender									
Male (referent)	62.3	1.00	ref	ref	ref	1.00	ref	ref	ref
Female	60.5	0.92	0.79	1.08	0.32	0.96	0.78	1.19	0.74
Age (years)					**0.012**				**0.008**
18–24 (referent)	63.9	1.00	ref	ref	ref	1.00	ref	ref	ref
25–39	56.5	0.73	0.52	1.01	0.055	0.67	0.44	1.02	0.064
40–54	61.6	0.91	0.66	1.25	0.56	1.04	0.69	1.59	0.84
55 and over	64.4	1.01	0.73	1.39	0.96	1.17	0.77	1.77	0.46
Annual income					**0.008**				**0.48**
Up to £15 000 (referent)	60.9	1.00	ref	ref	ref	1.00	ref	ref	ref
£15 001–£30 000	63.8	1.14	0.92	1.41	0.24	1.17	0.89	1.56	0.27
Over £30 000	62.6	1.08	0.88	1.33	0.46	1.14	0.86	1.51	0.36
Don’t know/prefer not to say	53.2	0.72	0.55	0.95	0.022	0.90	0.62	1.30	0.57

Models include 3279 observations from 1848 individuals. Bold font indicates significant associations (p<0.05).

LCI, Lower CI; UCI, Upper CI.

#### Use in smoke-free places

Support for use in smoke-free places was overall lower in 2014 (45.0%) than in 2013 (55.2%, figure 1 and table 4), and this remained significant in adjusted analyses. About 12% did not know whether to support or not (figure 1). Smokers were more likely than ex-smokers to support use in smoke-free places (table 4). The more experience respondents had with e-cigarettes, the higher the odds that they supported their use in smoke-free places. Respondents who knew that only a small portion of the health risks of smoking came from nicotine were more likely to support use in smoke-free places than those who thought that nearly all the risks came from nicotine, and those who perceived e-cigarettes to be less harmful than cigarettes were more likely to support use in smoke-free places. Compared with a low income, a high income was associated with less support of use in smoke-free places. Age and gender were not associated with support (table 4). There were no significant interactions between demographics and survey wave (see online supplementary table S1).

**Table 4:** Association between respondent characteristics and support for e-cigarette use in smoke-free places

		Bivariate				Adjusted/multivariable			
	Use should be allowed (%)	OR	95% LCI	95% UCI	p Value	OR	95% LCI	95% UCI	p Value
Wave									
2013 (referent)	55.2	1.00	ref	ref	ref	1.00	ref	ref	ref
2014	45.0	**0.69**	**0.62**	**0.76**	**<0.001**	**0.48**	**0.28**	**0.81**	**0.007**
Smoking status									
Ex-smoker (referent)	38.7	1	ref	ref	ref	1	ref	ref	ref
Current smoker	53.5	**1.73**	**1.44**	**2.07**	**<0.001**	**1.72**	**1.40**	**2.10**	**<0.001**
E-cigarette					**<0.001**				**<0.001**
Never tried (referent)	35.0	1.00	ref	ref	ref	1.00	ref	ref	ref
Tried, not using	52.4	**1.88**	**1.57**	**2.24**	**<0.001**	**1.89**	**1.57**	**2.29**	**<0.001**
Non-daily use	65.0	**2.90**	**2.40**	**3.51**	**<0.001**	**2.60**	**2.13**	**3.17**	**<0.001**
Daily use	73.5	**4.50**	**3.52**	**5.76**	**<0.001**	**4.29**	**3.29**	**5.60**	**<0.001**
Risks of smoking from nicotine					**<0.001**				**<0.001**
Nearly all (referent)	47.9	1.00	ref	ref	ref	1.00	ref	ref	ref
Much more than half	47.4	0.98	0.78	1.24	0.87	1.02	0.79	1.31	0.89
Around half	46.3	0.88	0.69	1.11	0.28	0.87	0.67	1.12	0.28
Some but well under half	52.2	1.06	0.83	1.35	0.64	0.99	0.76	1.29	0.93
None/very small	70.1	**1.95**	**1.46**	**2.62**	**<0.001**	**1.74**	**1.27**	**2.39**	**<0.001**
Don’t know	45.2	0.91	0.68	1.22	0.55	1.08	0.78	1.48	0.65
Relative harm of e-cigarettes									
Not less harmful	34.1	1.00	ref	ref	ref	1.00	ref	ref	ref
Less harmful	60.8	**2.46**	**2.13**	**2.83**	**<0.001**	**2.00**	**1.71**	**2.33**	**<0.001**
Gender									
Male (referent)	50.2	1.00	ref	ref	ref	1.00	ref	ref	ref
Female	51.5	1.05	0.89	1.23	0.58	0.93	0.77	1.14	0.49
Age (years)					0.13				**0.037**
18–24 (referent)	51.6	1.00	ref	ref	ref	1.00	ref	ref	ref
25–39	47.4	0.83	0.60	1.15	0.26	0.80	0.54	1.19	0.27
40–54	51.1	0.96	0.70	1.31	0.78	0.97	0.66	1.41	0.86
55 and over	52.4	1.04	0.73	1.38	0.98	1.00	0.68	1.45	0.98
Annual income					**0.038**				0.073
Up to £15 000 (referent)	53.8	1.00	ref	ref	ref	1.00	ref	ref	ref
£15 001–£30 000	52.9	0.95	0.76	1.18	0.63	0.89	0.69	1.16	0.39
Over £30 000	48.1	**0.78**	**0.63**	**0.97**	**0.025**	**0.70**	**0.54**	**0.91**	**0.007**
Don’t know/prefer not to say	46.2	**0.73**	**0.54**	**0.98**	**0.035**	0.75	0.53	1.06	0.10

Models include 3279 observations from 1848 individuals. Bold font indicates significant associations (p<0.05).

LCI, Lower CI; UCI, Upper CI.

## Discussion

Overall, in this sample of smokers and ex-smokers, the majority did not support very restrictive e-cigarette policies, such as making e-cigarettes less available than traditional tobacco cigarettes, bans on all e-cigarette advertising and bans on their use in smoke-free places. However, support for prohibiting e-cigarette use in smoke-free places increased, and in 2014, very similar proportions supported and opposed a ban. To a lesser extent, support for banning advertising also increased over the study period.

Support for policies was related to misperceptions about nicotine harms and the relative harmfulness of electronic and traditional tobacco cigarettes; those with less accurate perceptions were more likely to support more restrictive policies. Respondents with experience of e-cigarettes were more likely to support less restrictive policies than those who had never tried e-cigarettes; support appeared to be highest among regular users. Current smokers were more supportive of use of e-cigarettes in smoke-free places and unrestricted advertising than ex-smokers, but similarly supportive of equal or higher availability of e-cigarettes relative to cigarettes.

This study provides an initial insight into public support of potential key regulatory approaches to e-cigarettes among smokers and ex-smokers. The study findings have to be considered in the light of some limitations. To the best of our knowledge, this is the first time support for availability relative to cigarettes has been assessed. Future studies may benefit from refined questions that could break down availability into more detailed questions (eg, types of shops, by prescription); the advertising question could be improved by specifying advertising channels. Attitudes towards banning e-cigarette use are also likely to vary depending on the location (eg, outdoor vs indoor and workplaces vs restaurants^29^). It is also unlikely that the survey assessed all factors that may affect policy support; in addition to a respondent's own smoking and e-cigarette use, that of family or peers may also affect support and general political beliefs (eg, more liberal or more authoritarian) may also affect support for regulation. The sample included only smokers and ex-smokers, while never-smokers make up over 50% of the British population.^35^ Results for never-smokers may differ; they may, for example, report higher levels of support for restrictions^15^ ^27^ ^28^ and less nicotine knowledge.^36^

Similar to the present findings, increase in support over time for more restrictive regulations has also typically been observed with smoke-free policies.^37^ And for smoke-free policies, support generally increases markedly once policies are introduced.^37^ ^38^

Comparison with levels of support for e-cigarette regulation found in previous surveys can only be tentative because of differences in samples and questions used. Despite possible differences in absolute levels of support however, the present and previous surveys^12^ ^26^ ^27^ ^29^ consistently (and unsurprisingly) find that those with e-cigarette experience are less likely to support restrictions.

The present and previous surveys also show consistency in finding that increased (inaccurate) perceived harm of e-cigarettes is associated with increased support for restrictive policies.^12^ ^26^ ^27^ This is pertinent because a number of studies have shown that most people are not aware of the limited role of nicotine as a cause of smoking-related morbidity and mortality. Around half of smokers and ex-smokers from a number of countries erroneously thought that nicotine causes most of the smoking-related cancers.^39^ Only about 15% of smokers in Sweden^40^ and Great Britain,^36^ and <10% of the general population in Great Britain,^36^ knew that nicotine is responsible for a small portion of the health harms of smoking. A very similar level of knowledge about nicotine harmfulness was found in the present survey. Misperceptions of nicotine may also be behind findings that about a third of past-year smokers in England believe that using nicotine replacement therapy for more than a year would be very or quite harmful to health,^41^ and may also be associated with increased perceived relative harm of e-cigarettes.^32^ Misperceptions of harm are of concern because of the associations between knowledge, harm perception and policy support.

Future research may explore what harms respondents attribute to nicotine; this may be addiction as well as more direct harm to health which the measure used here and in the previous studies^30^ ^34^ did not distinguish. There are many aspects related to e-cigarette policy where evidence is still lacking. Importantly, support of different policies by groups other than smokers and ex-smokers needs to be explored; this should include those who have never smoked and anyone exposed to e-cigarette emissions at work. Further scientific evidence, for example, on the effects of e-cigarettes on air pollution, or the effects of e-cigarette advertising on smoking cessation and uptake, would allow balancing of benefits and risks of potential policies to different groups of the population.

### Conclusions

Support for specific e-cigarette policies appeared to be associated with smoking status, e-cigarette use, knowledge about nicotine and perceived relative harm. Fewer restrictions on e-cigarette use in smoke-free places and advertising were more likely to be supported by current smokers, respondents with experience of e-cigarette use, respondents who perceived e-cigarettes to be less harmful, and those who were aware of the role of nicotine in smoking-related harms to health. Support for equal or higher relative availability of e-cigarettes relative to cigarettes was higher in respondents with experience of e-cigarette use, those who knew that most of the health harms of smoking are not due to nicotine, and those who perceived e-cigarettes to be less harmful than cigarettes.

**What this paper adds**

- This study shows the level of support among smokers and ex-smokers in Great Britain for equal or greater availability of e-cigarettes relative to cigarettes, allowing e-cigarette advertising and use in smoke-free places.
- Support for prohibiting e-cigarette use in smoke-free places increased, and in 2014, very similar proportions (over 40%) supported and opposed a ban. To a lesser extent, support for banning advertising also increased over the study period.
- Equal or higher availability relative to cigarettes was more likely to be supported by e-cigarette users, those who perceived e-cigarettes as less harmful than cigarettes, and those who knew that most of the health harms of smoking are not due to nicotine.
- Less restrictive policies for advertising and use in smoke-free places were more likely to be supported by smokers, e-cigarette users, those who perceived e-cigarettes to be less harmful than cigarettes and those who knew that only a small portion of the health harms of smoking are due to nicotine.
- These survey findings suggest that, in addition to smoking and e-cigarette use status, accurate knowledge about nicotine and perception of harm are determinants of policy support.
