# Supplementary material for: Support for e-cigarette policies: a survey of smokers and ex-smokers in Great Britain
Source: Tob Control. 2016 Jun 16;26(e1):e7–e15. doi: 10.1136/tobaccocontrol-2016-052987 (PMC5739866; doi:10.1136/tobaccocontrol-2016-052987)
Supplement: Supplementary table S1 — Interactions [file tobaccocontrol-2016-052987supp001.pdf]

Table S1: Interactions

	Availability				Advertising				Use in smoke-free places			
	**OR**	**95% CI**	**95% CI**	**p**	**OR**	**95% CI**	**95% CI**	**p**	**OR**	**95% CI**	**95% CI**	**p**
Wave * gender (Wave 3*female)	0.66	0.47	0.94	0.019	0.94	0.70	1.26	0.67	1.13	0.90	1.43	0.30
Wave * age												
Wave 3 * 25-39	1.32	0.62	2.83	0.47	1.35	0.74	2.45	0.33	1.24	0.72	2.11	0.44
Wave 3 * 40-54	0.99	0.47	2.07	0.98	0.89	0.50	1.59	0.70	1.33	0.80	2.21	0.27
Wave 3 * 55 and over	0.87	0.42	1.80	0.71	0.96	0.54	1.69	0.88	1.56	0.94	2.58	0.087
Wave * income												
Wave 3 * £15001 to £30000	1.07	0.67	1.70	0.79	0.80	0.55	1.17	0.25	0.92	0.67	1.27	0.63
Wave 3 * over £30000	0.96	0.61	1.50	0.86	0.75	0.51	1.09	0.13	1.10	0.81	1.50	0.54
Wave 3 * don't know/prefer not to say	0.96	0.54	1.67	0.88	0.87	0.50	1.49	0.60	1.22	0.79	1.86	0.37
